# Calibrating Low-Cost Smart Insole Sensors with Recurrent Neural Networks for Accurate Prediction of Center of Pressure

**DOI:** 10.3390/s24154765

**Published:** 2024-07-23

**Authors:** Ho Seon Choi, Seokjin Yoon, Jangkyum Kim, Hyeonseok Seo, Jun Kyun Choi

**Affiliations:** 1 Department of Artificial Intelligence and Robotics, Sejong University, Seoul 05006, Republic of Korea; choihoseon@sejong.ac.kr; 2 Department of Software, Sejong University, Seoul 05006, Republic of Korea; seokjin1013@gmail.com; 3 Department of Artificial Intelligence and Data Science, Sejong University, Seoul 05006, Republic of Korea; 4 School of Electrical Engineering, Korea Advanced Institute of Science & Technology, Daejeon 34141, Republic of Korea; jkchoi59@kaist.edu

**Keywords:** ground reaction force, center of pressure, FSR sensors, supervised learning, gait analysis

## Abstract

This paper proposes a scheme for predicting ground reaction force (GRF) and center of pressure (CoP) using low-cost FSR sensors. GRF and CoP data are commonly collected from smart insoles to analyze the wearer’s gait and diagnose balance issues. This approach can be utilized to improve a user’s rehabilitation process and enable customized treatment plans for patients with specific diseases, making it a useful technology in many fields. However, the conventional measuring equipment for directly monitoring GRF and CoP values, such as F-Scan, is expensive, posing a challenge to commercialization in the industry. To solve this problem, this paper proposes a technology to predict relevant indicators using only low-cost Force Sensing Resistor (FSR) sensors instead of expensive equipment. In this study, data were collected from subjects simultaneously wearing a low-cost FSR Sensor and an F-Scan device, and the relationship between the collected data sets was analyzed using supervised learning techniques. Using the proposed technique, an artificial neural network was constructed that can derive a predicted value close to the actual F-Scan values using only the data from the FSR Sensor. In this process, GRF and CoP were calculated using six virtual forces instead of the pressure value of the entire sole. It was verified through various simulations that it is possible to achieve an improved prediction accuracy of more than 30% when using the proposed technique compared to conventional prediction techniques.

## 1. Introduction

As consumer interest in health has grown, the ability to analyze and evaluate a person’s movement and body balance has become increasingly important [1,2]. Various devices to monitor a human body’s balance (e.g., sensors equipped with gait analyzers, wearable devices with accelerometers and gyroscopes, etc) have been developed for medical purposes [3,4]. However, such medical devices are limited in that they have difficulty continuously monitoring the wearer’s body balance in real time, and they are expensive to own personally. Alternatively, smart insoles are considered to be suitable for monitoring human body balance due to their price and versatility.

In a smart insole, the ground reaction force (GRF) and center of pressure (CoP) are key indicators that can be used to assess an individual’s health status and physical balance [5,6,7]. To detect the GRF and CoP values, various sensors are integrated into the smart insole. However, there are several challenges that make it hard to monitor such data directly [8]. First, these technologies require expensive equipment and sensors, which make them difficult to implement in medical institutions or research environments. The most widely used F-Scan system costs over USD 20,000, and it requires additional modules and provided software to function [9]. This makes them challenging to integrate and use with other embedded systems. Accordingly, these systems are optimally suited for use only in laboratory environments and are less suitable in real-world settings. This inevitably hinders the commercialization and widespread adoption of healthcare or wearable systems that require the measurement of GRF or COP.

To solve these issues, several studies have developed low-cost insole sensors, with advances in hardware as well as software [8,10,11]. For the hardware, various approaches have tried to insert small pressure sensors based on inexpensive materials, to address the high cost of the devices [10,12]. Most of these approaches have been designed in a form that calculates GRF and CoP during a gait cycle using load cells or force-sensitive resistor (FSR) sensors inserted in the smart insole. Since each FSR sensor costs only a few tens of dollars, even with 10 sensors, the total cost is only 1/10th of the price of the F-Scan system. This advantage addresses financial concerns and makes it easier to integrate with other systems because it can work with various microcontrollers.

For these low-cost sensors, the ability to predict the quality of the GRF and CoP data calculated by small pressure sensors increases as the number of sensors increases. However, this approach also raises the price of the entire system. For this reason, the primary effort on the hardware side is currently focused on developing sensors with cheaper materials.

Because it increases the cost of the smart insole, installing additional pressure sensors is not reasonable, as mentioned before [13,14]. Therefore, studies of ways to achieve better prediction performance using a limited number of sensors with the prediction algorithms are being conducted [15,16,17]. In [15], Lee et al. proposed an algorithm based on a neural network to estimate the knee and ankle angles based on an exoskeleton robot. With the proposed robot and prediction algorithm, the authors showed that it is possible to predict the angles of various joints. In [16], Oubre et al. introduced a novel data analysis method to predict GRF and CoP values using a low-cost wearable device. Here, the authors presented a method to accurately predict values using inaccurate FSR responses. There was also a study that developed a deep learning model to predict a three-dimensional GRF value using three single-axis load cells, in [17]. In the paper, Kim et al. showed that it was possible to predict GRF values in an outdoor environment using the proposed prediction algorithm. Such studies supplement the prediction accuracy of conventional limited low-cost sensors using various neural networks. However, these conventional studies do not include a proper data pre-processing scheme and did not evaluate whether the neural network models used were suitable for the given environment.

Accordingly, when developing a prediction algorithm specialized for a targeted device, a suitable neural network model and pre-processing technique are essential. In this paper, we propose algorithms for cases where the user’s data from the smart insole is sufficient, or insufficient, by selecting a prediction model for each algorithm that can maximize GRF and CoP prediction performance. The specific contributions can be summarized as follows.

- In various conventional studies GRF and CoP values are monitored using expensive equipment. This makes it hard for people to gather such data to check their health condition, and determine their center of gravity. To address this issue, we introduce a cost-effective GRF and CoP monitoring solution using low cost FSR sensors. This approach not only ensures the accuracy of the GRF and CoP values, but also enhances the accessibility of smart insole devices in medical environments.
- In conventional studies, the prediction model uses a simple perceptron model that has limited ability to reflect tendencies in the time series data [8]. To reflect user movement in the smart insole, the conventional research augments the input dimension before training the model. However, this approach makes it difficult to converge on optimal parameters and to analyze periodicity. To address this, we use a recurrent neural network model to capture sufficient information for the prediction, with only small-dimensional input data and identify longer periodicity.
- Generally, the prediction performance for a smart insole will vary depending on the type of neural network and pre-processing methods [18,19]. To address this issue, we analyze the characteristics of the data to determine the proper prediction methods. In addition, by comparing the prediction results with different neural network models and pre-processing schemes, we demonstrate that the proposed approaches are most suitable for a given device.

The structure of this paper is organized as follows. We review existing research in Section 2. Next, Section 3 provides an overall system model that covers the data collection and analysis methods. Section 4 presents various numerical results to demonstrate the effectiveness and suitability of the proposed scheme. The paper concludes with Section 5, which summarizes our research.

## 2. Related Works

As people’s interest in health increases, the importance of smart insoles has also gradually increased [20]. Various attempts have been made to identify inferring key factors that can be utilized to analyze a user’s body characteristics using low-cost sensors. Representative studies on this field are summarized below.

As shown in Table 1, most conventional approaches used low-cost FSR sensors and neural networks to predict the CoP value based on data acquired in a limited environment. Additionally, these approaches assume that sufficient data will be available in all situations and do not address the problem of securing a sufficient amount of data. To solve such issues, we consider both static and dynamic postures of the user and present various prediction methods depending on the amount of data.

## 3. System Model

**Figure 1:**
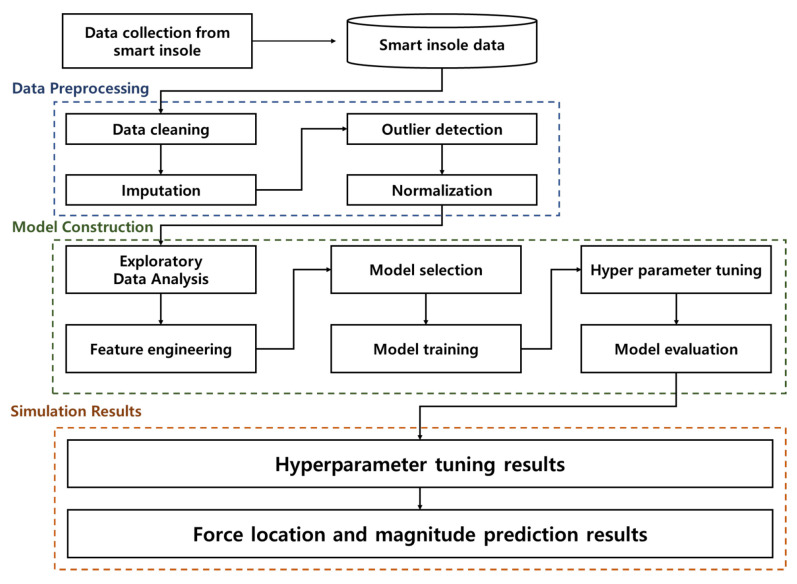
System model of the proposed GRF and CoP prediction scheme.

As depicted in Figure 1 we developed an artificial neural network to predict GRF and CoP through a smart insole consisting of only low-cost FSR sensors without F-Scan equipment. Initially, we conducted a comprehensive data collection process and proper cleaning using feature engineering techniques. In the data pre-processing, we employed various scaling and imputation processes considering the distribution of a given dataset. In this study, the most appropriate pre-processing scheme was chosen to complement the architecture of our deep learning model, aiming to minimize the L2 loss function effectively. The selection of the scheme was accomplished by simulating various scenarios to determine the most fitting structural configuration.

### 3.1. Acquisition of Data

**Figure 2:**
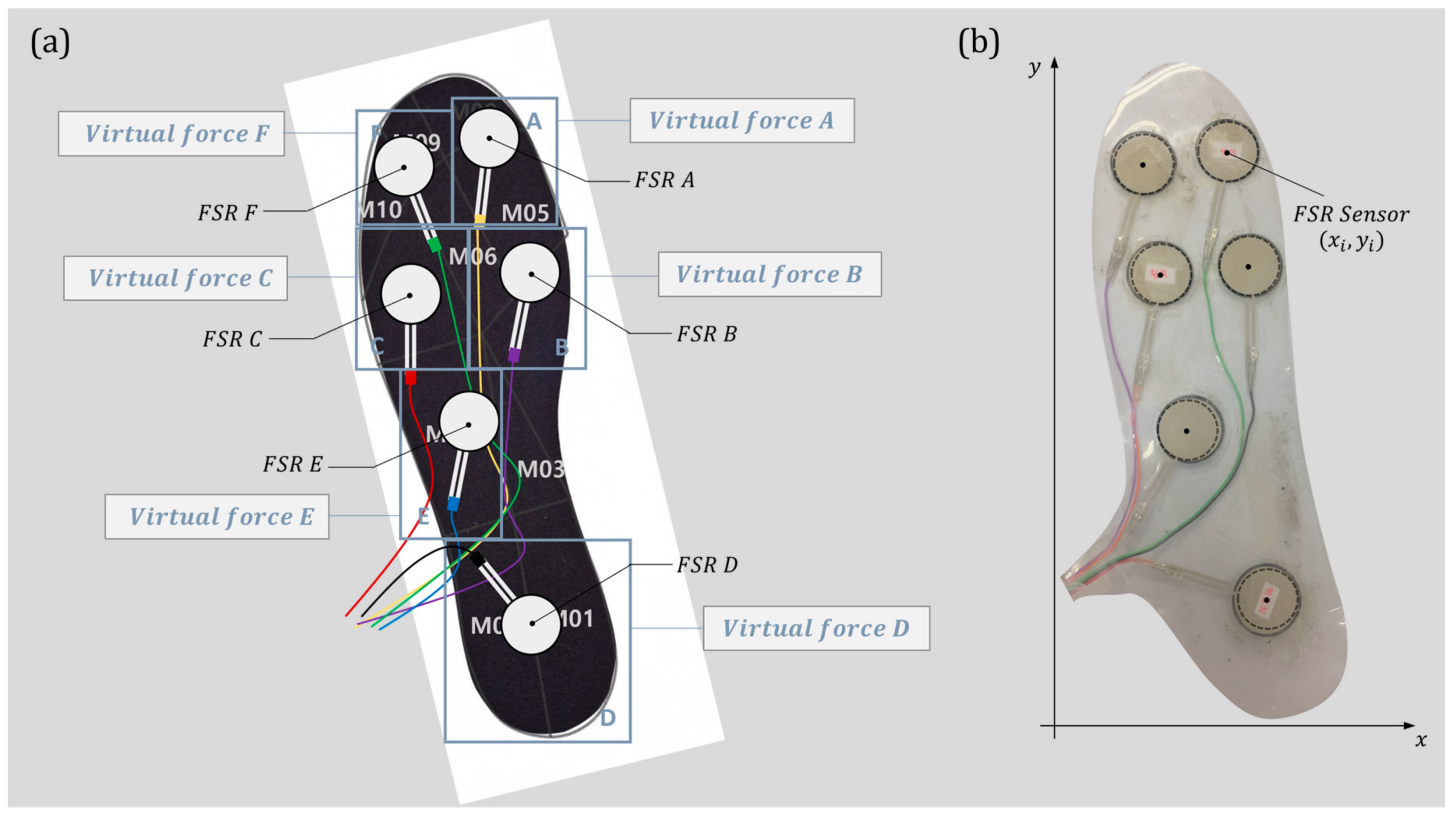
(**a**) Configuration of a low-cost insole sensor and its elements. (**b**) Fabricated low-cost insole sensor and its coordinate system.

We needed experimental data to train certain algorithms which have in/output values, so we conducted experiments with both an F-Scan system and a developed low-cost insole sensor. The F-Scan system is a commercial sensor with a lot of tiny pressure sensors so that precise pressure distribution and the following center of pressure can be measured with it. Our goal is to develop an algorithm that would provide a low-cost sensor with the same measurement performance as commercial sensors, so the data acquired from the F-Scan system could be used as the output values of the developed algorithm. We developed a low-cost insole sensor with six FSR sensors as shown in Figure 2. A detailed description is provided in our previous study [8]. The data acquired by this low-cost sensor would be the input values of our algorithms.

Our algorithm was developed to increase the overall GRF and CoP prediction performance, by predicting the pressure distribution around each FSR sensor using the measured values of the six FSR sensors. Because this is difficult to achieve with information just from the areas that can be measured by the FSR sensors, we defined further expanded areas beyond those, as shown in Figure 2a. We tried to predict information in new areas using the data measured by the FSR sensors. And we decided to use the pressure distribution in those areas by converting it into a virtual force that can be expressed as a single quantitative value [8]. In this way, we can predict GRF by simply adding up the virtual forces.

We also created moving coordinates for those virtual forces to predict the COP using a weighted mean approach calculation method. After specifying the area of virtual force and moving coordinates around each sensor, the force value could be derived from the pressure distribution within that range, and the coordinates x and y of that force are constructed as shown in Figure 2b.

**Figure 3:**
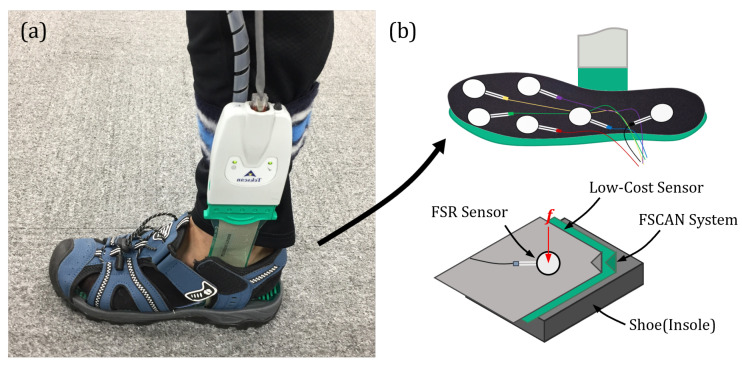
(**a**) Configuration of experimental setup, with the subject wearing an F-Scan system and the low-cost insole sensor, simultaneously. (**b**) The principle of data acquisition, which contains input (FSR sensor)/output (F-Scan) values for training the algorithm.

In order to learn a model that predicts F-Scan values using data from the six FSR sensors, an experimental environment was constructed and data were collected as follows. We recruited eight participants and conducted several experiments for data acquisition. The experiment subjects wore both the F-Scan and low-cost insole sensors simultaneously, as shown in Figure 3. Those experiments consisted of several motion tasks, like scan, gait (1 and 2 km/h) on the treadmill, squat, and sit-to-stand, while simultaneously wearing both the F-Scan system and low-cost insole sensors, as shown in Figure 3.

The scan task involves standing still on two feet and tilting the body to rotate the center of pressure counter-clockwise and back and forth. This task is performed to add information to the model about the pressure distribution under the foot according to while shifting the center of gravity in various situations with the foot on the ground. Except for the scan task, the remaining four movements are not only the most frequently performed movements in daily life, but also are the most frequently used in rehabilitation processes. Sit-to-stand and gait are the most commonly used movements in daily life, and squats are often used to align joints and train muscles to improve body stability. These tasks were selected because the low-cost insole sensor and our developed model can be used in healthcare applications that are most likely to employ wearable sensors for rehabilitation or daily life assistance.

In such experimental environments, data were collected from various experimental groups. Each of the experimenters performed the following five actions: (1) standing on both feet and moving the center of mass counterclockwise; (2–3) walking at speeds of 1 km/h and 2 km/h; (4) squatting 10 times; and (5) sitting down and standing up 10 times. The specific details of the experiment subjects are as follows.

Using the data described in Table 2, the algorithm presented in this paper was applied to determine the optimal prediction method according to the integration and management of each data set. The performance improvement results for each case were then compared with those from the conventional method.

### 3.2. Data Pre-Processing

In this paper, we utilized pressure data from the five distinct actions performed by the six subjects outfitted with an F-Scan device and FSR sensors, as depicted in [8]. The data measured by the F-Scan were organized with the six virtual force data and twelve location datapoints. Subsequently, we conducted a supervised learning process to predict these eighteen F-Scan features using the pressure values measured by the six individual FSR sensors.

#### 3.2.1. Imputation

In our approach, we attempted to construct a neural network model to predict the GRF and CoP values. A neural network can be conceptualized as a function composed of sequential matrix operations. Since missing values make it hard to train the model properly, we employed an imputation scheme to substitute any missing value with appropriate data. Although there are various types of imputation methods that can substitute missing values (i.e., linear interpolation, KNN, spline values), the absence of time series characteristics in missing data restricts the use of complex mechanisms [26]. Therefore, the mean or median of the dataset were utilized to fill the missing data in this paper.

#### 3.2.2. Scaling

AI models typically utilize various types of input, so the model’s input data has different units and numerical ranges depending on each type of data. For example, according to the data collected in this study, the average of the virtual force was 241.637 and the value of position was 9.107. As a result, the raw data is not evenly distributed across the entire numerical space and is biased, making model convergence difficult. To improve the convergence of the model, it is essential process to ensure that the data are distributed evenly across the entire numerical space, through scaling.

Generally, a scaler is an algorithm that normalizes the distribution of input data [27]. Here, a standard scaler, min–max scaler, and robust scaler were used in our domain. These are mathematically formulated as

(1) $$\begin{array}{cc} \mathrm{Standard}\;\mathrm{scaler}:\;\;x_{i} & =\frac{x_{i}-\mu_{i}}{\sigma_{i}}\\ \mathrm{Min}\;\max\;\mathrm{scaler}:\;\;x_{i} & =\frac{x_{i}-x_{min,i}}{x_{max,i}-x_{min,i}}\\ \mathrm{Robust}\;\mathrm{scaler}:\;\;x_{i} & =\frac{x_{i}-Q_{2,i}}{Q_{3,i}-Q_{1,i}}. \end{array}$$

In Equation (1), for the *i*th feature $\mu_{i}$ refers to the mean, $\sigma_{i}$ is the standard deviation, $x_{min,i}$$x_{max,i}$ are the minimum and maximum values, respectively. In addition, $Q_{1,i}$, $Q_{2,i}$ and $Q_{3,i}$ are the first, second and third quartiles. Since the performance of a neural network depends on the type of scaler, we applied various scalers in the simulation Section to find the most suitable scaler for the model.

#### 3.2.3. Feature Engineering

As depicted in the previous Section, we predicted GRF and CoP values using the data from low-cost FSR sensors. The six points of FSR data (i.e., which were measured from different states of a person) were used as the input data. When predicting the size of the virtual force, the height of the person should be reflected in the prediction process. However, we needed to focus on the pattern of change in the measurement values, regardless of the height of the person, when predicting the coordinates of the FSR data. Therefore, an existing FSR sensor was used to predict the virtual force value, and a new feature was created to predict the coordinates of the virtual force.

### 3.3. System Model to Predict the CoP Value

The main purpose of this paper is to try and select a suitable prediction structure and neural network model to achieve accurate CoP prediction results. Therefore, applicable prediction methods were proposed based on the characteristics and properties of a given data set. By comparing the accuracy of various prediction results, the most appropriate scheme was selected for implementation in a smart sensor device.

#### 3.3.1. Various Structures of CoP Prediction

**Figure 4:**
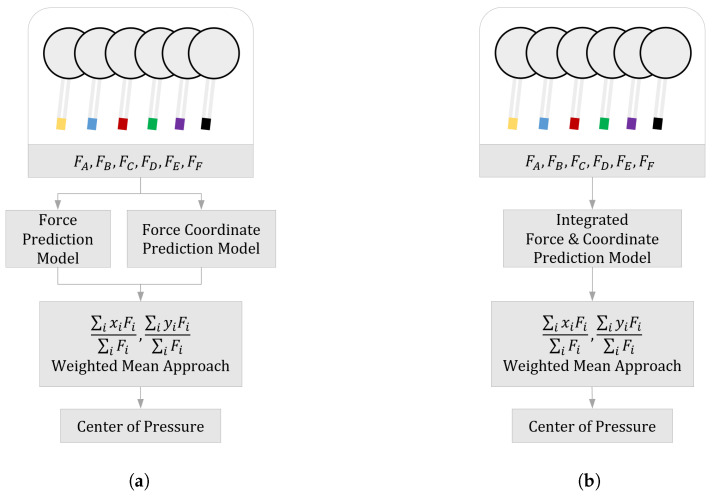
Various approaches to predict user movement. (**a**) Specialized method to predict virtual forces and coordinates; (**b**) integrated method to predict virtual forces and coordinates.

As shown in Figure 4, we propose two prediction structures to determine the CoP value. The method in Figure 4a deals with a structure for predicting the coordinates and force of a certain location by developing a specialized prediction model. By developing 12 specialized prediction models, it is possible to calculate the CoP value using the predicted data. The method n Figure 4b calculates the CoP by developing a single structure that predicts virtual forces and coordinates simultaneously. Using this approach, we can compare the effectiveness of each method, and select a technique suitable for the actual environment by proposing various structures.

#### 3.3.2. Long Short-Term Memory

To predict force and coordinate values, long short-term memory (LSTM) can be used as one of the models to achieve accurate predictions [28,29]. Since the data acquired from the smart insole are time-series-based, a recurrent neural network (RNN) series model, which stores information about previous inputs and uses it to infer outputs, is appropriate in our situation. However, RNNs have a vanishing gradient problem, which results in them losing information from long past periods due to the overlapping and multiplication of model parameters. LSTM was designed to solve the RNN’s long-term dependency problem by introducing a cell state mechanism. Given the LSTM’s characteristic ability to maintain important information for extended periods while discarding irrelevant data, it can be suitably applied to the problem of predicting the value of each variable from the smart insole sensor at future points. Therefore, we chose to use LSTM as one of the representative models to achieve prediction results.

#### 3.3.3. CNN-LSTM to Predict Parameters

The LSTM presented in the above Section is a powerful model for processing time series data. However, it is limited by its high computational complexity, and the information loss that occurs with long sequences of data. In addition, LSTM is hard to apply to high-dimensional data. To solve this issue, various studies were conducted that used CNN-LSTM for multidimensional time series data prediction problems [30,31,32]. CNN-LSTM is a neural network structure that can learn both spatial and temporal characteristics from time series data. Here, CNN is used as an encoder to reflect high-dimensional features, and LSTM is used as a decoder to generate time series data. In a typical CNN-LSTM configuration, the features of multidimensional data are extracted through a Convolutional Neural Network (CNN), and the extracted feature maps are converted to sequence data and transferred to the LSTM. Afterwards, the temporal characteristics of the sequence data are trained in the LSTM to achieve prediction results for a specific point in time.

The CNN-LSTM model is used in this paper for multidimensional time series data, so the model is implemented by applying a one-dimensional convolution neural network and then applying LSTM. Therefore, when the time series data pass through the CNN, the shape of the data is shown as follows:

(2) $$L_{out}=\left[\frac{L_{in}+2\times \mathrm{padding}-\mathrm{kernel}}{\mathrm{stride}}+1\right]$$

In Equation (2), the input and output should be identity mapped. Therefore, we set the values of the variables as padding=1, kernel=3, and stride=1, respectively.

## 4. Simulation Results

In this section, we present the prediction results calculated using our proposed algorithm. These results were obtained using the various algorithms mentioned in Section 3.3, utilizing sensor data from a smart insole. The data for this study were collected from seven individuals of different genders and ages, each wearing the smart insole. When constructing a prediction model, it is common practice to divide the data into training and test sets at a certain ratio. However, when the data volume is relatively small, the prediction results can be biased. Therefore, we also examined a case that designated the data from six individuals as the training set and the data from the remaining individual as the test set.

The main purpose of the entire simulation is to estimate the CoP (center of pressure) value of the smart insole at a future time period. To accomplish this, it is essential to estimate both the magnitude and the coordinates of the virtual force using the proposed methods. Here, we propose two different methods to predict variables: (i) a specialized method to predict each variable, and (ii) predicting variables through one integrated method. In the specialized model, it is possible to construct an individual prediction model and optimize it according to the unique characteristics of the specific data, which is expected to maximize prediction accuracy. Also, integrated methods have the advantage that they are able to learn complex interactions among various variables and show efficient performance in terms of entire system. Accordingly, we compare the performance of these models and indicate which model is more appropriate for predicting the user’s CoP value.

In order to evaluate the performance of the proposed prediction methods, prediction error was measured using the Root Mean Square Error (*RMSE*) value [33] which is mathematically formulated as

(3) $$RMSE=\sqrt{\frac{1}{N}\sum_{t=1}^{N}{\left(o_{t}-{\tilde{o}}_{t}\right)}^{2}}.$$

In Equation (3), *N* means the number of sampling points. Also, $o_{t}$ and ${\tilde{o}}_{t}$ refer to the actual and predicted outputs at time *t*, respectively.

### 4.1. Hyper-Parameter Setting

Since various models and techniques are used for the comparative accuracy analysis, it is necessary to optimize the hyper-parameters in the simulation for each model, and compare the corresponding accuracy. In our paper, optimization was performed separately for the two cases (e.g., the specialized method and integrated method). In order to select the proper model to be used for each method, the artificial neural network (ANN) technique used in [8] was simulated to set the baseline model. In addition, the LSTM and CNN-LSTM models were simulated as the proposed models. By optimizing all of the hyper-parameters, it is possible to select the appropriate method and models for predicting the CoP value. Here, the hyper-parameter optimization was achieved using the Asynchronous Hyperband Scheduler. The algorithm executes various hyper-parameters in parallel, and terminates low-performing trials early, then allocates resources to explore the hyper-parameters of high-performing trials. Such a mechanism is suitable for this study, which requires the optimization of multiple models, and the hyper-parameters were optimized by the scheduler with 100 trials for each case.

The parameter search space consists of the type of imputer (e.g., mean, median), type of scaler (e.g., standard scaler, min–max scaler, robust scaler), type of optimizer (Adam, NAdam, Adagrad, RAdam, SGD), and learning rate. The search space is further tailored depending on the model structure. In the optimization process, parameters such as the number of layers and hidden size are also considered.

### 4.2. Determining the Proper Data Pre-Processing Methods

Before proceeding with the prediction results for CoP, we selected the proper data pre-processing scheme using the quantitative simulation results.

**Figure 5:**
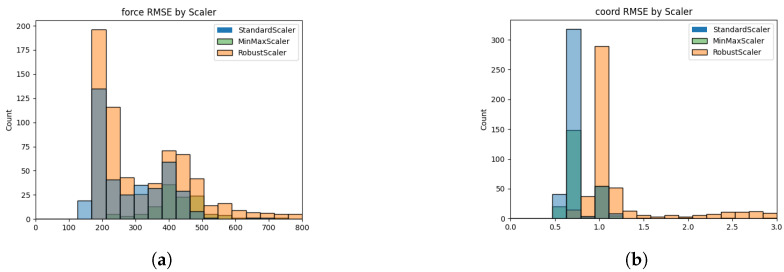
Accuracy comparison to determine proper imputation scheme. (**a**) Imputation results for virtual force; (**b**) imputation results for coordinate value.

In Figure 5, we show a histogram plot that represents the count of trials depending on different scaling methods in the overall prediction methods. For the virtual force prediction in Figure 5a, standard scaler and robust scaler are suitable because they exhibited relatively low RMSE values in most trials. When predicting the coordinate value in Figure 5b, standard scaler and min–max scaler were determined to be suitable for application in the scaling process, as theprediction error was relatively low. Based on the results, the standard scaler was judged to be suitable for scaling the data set.

### 4.3. Prediction Performance for Virtual Forces

In this section, we analyze the virtual force prediction performance for various prediction methods and models. With this approach, it is possible to select the most appropriate technique for predicting virtual force.

In Table 3, we show the prediction accuracy of the different techniques that we mentioned in Section 3.3.1. In the table, Case 1 refers to the case where the training and test data are divided into a specific ratio. Also, in Case 2, the data from the six experimenters are used as training data, and the data of one other experimenter are set as the test data. Further, simulations were conducted on two prediction methods: the specialized method, which develops a specialized prediction model for each variable, and the Integrated method, which makes predictions through one integrated model.

In Case 1, the prediction model is developed by collecting the entire data set, and a specialized model for each variable is constructed based on the LSTM which shows the highest accuracy. The results confirmed that the LSTM series is more suitable for increasing prediction accuracy than using the ANN series model, due to the continuity of the data. Also, in Case 2, where the training and test data are divided based on the user, it was found that predicting the virtual force using a CNN-LSTM model is appropriate. From the results, it can be determined that the CNN-LSTM model was able to extract key feature points better than the LSTM model alone. We also checked the prediction results for coordinate values to determine the proper method and model for predicting the CoP value.

Examining the case of LSTM, which demonstrated the highest accuracy in predicting virtual force, we confirmed that performance was improved by more than 18.9% using the specialized method instead of the Integrated method.

### 4.4. Prediction Performance for Coordinates of Virtual Force

In this section, we compare the techniques for predicting the coordinate values of each virtual force, which is one of the important factors for calculating the center of pressure (CoP). The techniques for splitting the training and test data were divided into two cases, as in the previous prediction. Additionally, three types of prediction models were constructed: ANN, LSTM, and CNN-LSTM.

In Table 4, it is possible to review the analysis results for the most appropriate prediction methods and models based on the amount of data available. When there is a sufficient amount of collected data, integrating the entire dataset without differentiating based on user characteristics (e.g., age, gender, body type) proves to be cost-effective. In Case 1, developing a specialized model for each variable proves to be the most effective approach. Notably, the most successful model, which utilizes a CNN-LSTM configuration in the specialized case, can enhance prediction accuracy by up to 42.5% compared to the least effective scenario, which employs an ANN model [8] in the integrated case, as shown in the table.

In Case 2, where there is a lack of substantial initial user data, the specialized method is preferable for improving prediction accuracy. The results demonstrated that overall performance is improved by more than 33% when using the specialized method instead of the Integrated method. However, there was no significant difference in accuracy between different model types within the specialized case.

Based on the results, we confirm that using the specialized method for the overall dataset can improve prediction accuracy. Additionally, the results indicate that the LSTM model, which focuses on continuous tendencies, is appropriate for predicting virtual force. Furthermore, the CNN-LSTM model, which incorporates the characteristics of various spatial variables, is the most suitable for predicting coordinate values.

### 4.5. Analysis of CoP Prediction Based on Selected Techniques

Next, we would like to analyze the CoP prediction accuracy using the proposed specialized method and neural network model.

**Figure 6:**
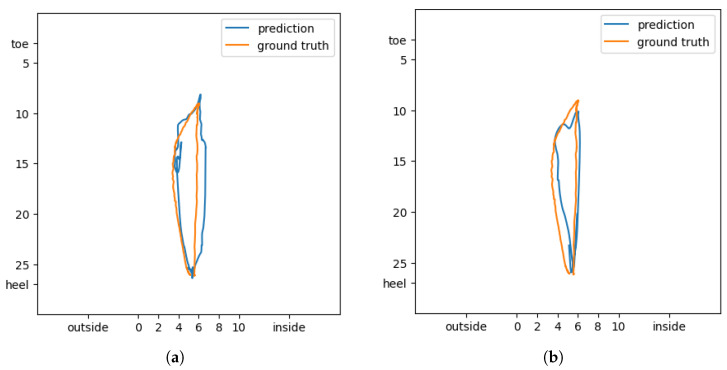
Comparison of CoP prediction according to different prediction methods and models. (**a**) CoP prediction results using specialized methods with optimal models; (**b**) CoP prediction results using integrated methods with optimal models.

In Figure 6, we compare the CoP prediction performance results when using the specialized method and the Integrated method. In the figure, we can check important factors to determine whether the prediction results cover the value of Ground truth or not. In Figure 6a, it is possible to check that most of the movement range of CoP is covered when using the corresponding prediction technique. However, a large prediction error appears in the left part of Figure 6b. Through the results, it is confirmed that the specialized methods with optimal models are more suitable for predicting CoP values.

## 5. Conclusions

This study investigated techniques for predicting CoP, an important indicator for assessing a user’s health and postural stability. The results showed that when the dataset acquired from the smart insole is sufficient, predicting the CoP through an integrated neural network model is cost-effective. However, in environments where the collected data are limited, developing a specialized model for each variable is the best way to achieve more accurate CoP values. Using various simulations, it was confirmed that prediction accuracy could be improved by up to 18.9% for virtual forces and up to 33% for coordinate values by using the specialized method instead of the Integrated method, with the optimal model used in each case.

In summary, this paper argues that it is possible to achieve the predicted CoP value using the proposed technique, which sufficiently covers the indicators derived from the conventional ground truth value. Also, various simulation results showed that the proposed prediction technique can stably cover the user’s CoP value. This work sets the stage for intriguing future directions in this research, including the following.

- With the development of a lightweight application, we aim to derive analysis results from data acquired in various external user environments.
- By linking with additional wearable devices, we plan to conduct research on how to maximize prediction performance for changes in the user’s CoP according to surrounding environmental indicators.
- It may be possible to analyze the CoP values of patients with dementia and other diseases using devices and applications, to achieve meaningful insights from the results.

## Figures and Tables

**Table 1:** Related works on predicting CoP values using various IoT sensors.

Study	Description and Pros	Cons
Chou et al. [21]	Calculate the trajectory of CoP using a weighted average approach in a smart insole with 89 pressure sensing points and evaluate its accuracy.	The analysis method was too simple; it was not verified that the proposed method sufficiently reflected the entire complexity of the foot pressure distribution.
Moon et al. [22]	The center of gravity was predicted using data collected from smart sensors and the Bi-LSTM model, with an experimental group consisting of 15 healthy young men and 15 elderly men, which was larger than conventional studies.	This method was primarily tailored to walking experiment conditions, and it was necessary to verify whether the proposed model can be applied to various daily activities.
Tan et al. [23]	Designing a low-cost, high-resolution smart insole using fractal dimensional analysis techniques to detect and analyze the center of pressure (CoP).	The study primarily measured CoP changes while standing, and further research is needed to verify its applicability to various daily activities.
Duong et al. [24]	Developing a recurrent neural network with a Bi-LSTM structure to estimate the COP trajectory based on data from the insole sensor.	Focusing on the task of walking on flat ground, and it is necessary to verify whether the proposed model is applicable to various user movement environments.
Hu et al. [25]	Designing a smart insole based on low-cost pressure sensing resistors and proposed AI model to estimate the CoP value.	Since authors mainly focused on static postures and sitting-to-standing transitions, further researches is needed to confirm applicability to variety of activities in daily life.

**Table 2:** Physical information of the experimenters.

Subject No.	Gender	Age	Height (cm)	Weight (kg)	Shoe Size
1	Male	26	179	67	US8
2	Male	27	180	74	US8
3	Male	24	173	71	US8
4	Male	24	185	77	US8
5	Male	32	173	72	US8
6	Male	34	171	70	US8
7	Male	38	173	79	US8
8	Male	35	169	74	US8

**Table 3:** Comparison of virtual force prediction accuracy using RMSE (N).

	Case 1		Case 2	
Methods	Specialized	Integrated	Specialized	Integrated
**ANN** [8]	200.127	200.373	184.365	177.361
**CNN-LSTM**	178.705	203.214	175.383	**171.708**
**LSTM**	**155.939**	192.334	188.68	179.106

**Table 4:** Comparison of virtual force coordinate prediction using RMSE (cm).

	Case 1		Case 2	
Methods	Specialized	Integrated	Specialized	Integrated
**ANN** [8]	0.655	0.943	**0.671**	0.974
**CNN-LSTM**	**0.542**	0.927	**0.678**	1.022
**LSTM**	0.586	0.901	**0.676**	1.005

## Data Availability

The data reflects the participants’ personal information, so it cannot be disclosed in response to any requests.
